# Dynamics of Glutamatergic Drive Underlie Diverse Responses of Olfactory Bulb Outputs *In Vivo*

**DOI:** 10.1523/ENEURO.0110-21.2021

**Published:** 2021-04-15

**Authors:** Andrew K. Moran, Thomas P. Eiting, Matt Wachowiak

**Affiliations:** 1 Interdepartmental Program in Neuroscience, University of Utah School of Medicine, Salt Lake City, UT 84112; 2 Department of Neurobiology and Anatomy, University of Utah School of Medicine, Salt Lake City, UT 84112

**Keywords:** active sensing, calcium, glutamate, imaging, sniffing, temporal coding

## Abstract

Mitral/tufted (MT) cells of the olfactory bulb (OB) show diverse temporal responses to odorant stimulation that are thought to encode odor information. Much of this diversity is thought to arise from inhibitory OB circuits, but the dynamics of excitatory input to MT cells, which is driven in a feedforward manner by sensory afferents, may also be important. To examine the contribution of excitatory input dynamics to generating temporal diversity in MT cells, we imaged glutamate signaling onto MT cell dendrites in anesthetized and awake mice. We found surprising diversity in the temporal dynamics of these signals. Inhalation-linked glutamate transients were variable in onset latency and duration, and in awake mice the degree of coupling to inhalation varied substantially with odorant identity and concentration. Successive inhalations of odorant produced nonlinear changes in glutamate signaling that included facilitating, adapting and suppressive responses and which varied with odorant identity and concentration. Dual-color imaging of glutamate and calcium signals from MT cells in the same glomerulus revealed highly correlated presynaptic and postsynaptic signals across these different response types. Suppressive calcium responses in MT cells were nearly always accompanied by suppression in the glutamate signal, providing little evidence for MT cell suppression by lateral or feedforward inhibition. These results indicate a high degree of diversity in the dynamics of excitatory input to MT cells, and suggest that these dynamics may account for much of the diversity in MT cell responses that underlies OB odor representations.

## Significance Statement

Temporal patterns of excitation and inhibition among olfactory bulb (OB) output neurons [mitral/tufted (MT) cells] play important roles in coding olfactory information. Using the glutamate sensor iGluSnFR expressed on MT cells, we found surprising diversity in the dynamics of their excitatory input: odorant-evoked glutamate signals varied within a respiratory cycle and across repeated samples of odorant, and varied with both odorant identity and concentration. Simultaneous imaging of presynaptic glutamate and postsynaptic calcium from MT cells revealed high correspondence in the dynamics of glutamatergic input and MT cell output. These results suggest that the dynamics of excitatory input alone can account for much of the diversity of MT cell responses that underlie odor representations *in vivo*.

## Introduction

The neural activity underlying odor perception is inherently dynamic. In vertebrates, olfactory sensory input arrives at the brain as bursts of activity driven by each inhalation, and patterns of inhalation are actively controlled by an animal as it samples its environment. At the level of the olfactory bulb (OB), olfactory sensory neurons (OSNs) expressing a single type of odorant receptor converge onto glomeruli (Vassar et al., 1994) where they make glutamatergic synapses onto targets including juxtaglomerular interneurons and the principal OB output neurons, mitral/tufted (MT) cells (Wachowiak and Shipley, 2006). Odorant responses among MT cells can be temporally complex, displaying sequences of excitation and suppression that occur relative to a single inhalation and across multiple inhalations (Chaput, 1986; Patterson et al., 2013; Díaz-Quesada et al., 2018; Eiting and Wachowiak, 2020). Olfactory information is likely represented both by the identity of activated MT cells as well as by their temporal dynamics (Chaput, 1986; Schaefer and Margrie, 2007; Wilson et al., 2017; Li et al., 2020).

Much of the complexity in MT cell response patterns has been attributed to processing by inhibitory OB circuits, in particular, inhibitory circuits that target the apical or lateral MT cell dendrites (Gire and Schoppa, 2009; Shao et al., 2012; Fukunaga et al., 2014; Geramita and Urban, 2017). Intrinsic properties of MT cells may also contribute to diversity in MT cell odorant response patterns (Balu and Strowbridge, 2007; Padmanabhan and Urban, 2010). However, excitatory synaptic inputs to MT cells, which occur solely on the MT cell apical tuft in each glomerulus, can also contribute to generating temporally complex and diverse patterns of MT cell spiking. OSNs themselves respond to odorant inhalation with different latencies and show nonlinear changes in their activation patterns as a function of odorant concentration, sampling time, sampling frequency, and behavioral state (Spors et al., 2006; Verhagen et al., 2007; Iwata et al., 2017; Ackels et al., 2020). Additional diversity in excitatory synaptic input to MT cells may arise from multisynaptic pathways, for example, via external tufted (ET) cells (De Saint Jan et al., 2009; Shao et al., 2012; Banerjee et al., 2015; Gire et al., 2019). Regardless of its source, the contribution of glutamatergic input dynamics to determining MT cell odorant response patterns *in vivo* remains poorly understood.

Here, to characterize the dynamics of glutamatergic input to MT cells, we imaged odorant-evoked and inhalation-linked glutamate signals in glomeruli of the mouse OB using the genetically-encoded glutamate sensor iGluSnFR and its second-generation variants (Marvin et al., 2013, 2018), expressed in the MT cell population or selectively in piriform-projecting MT cells or in superficial tufted cells (sTCs). Glomerular glutamate transients reported inhalation-linked dynamics with high fidelity, allowing temporally precise monitoring of glutamatergic signaling across the dorsal OB in both anesthetized and awake mice. We observed a striking degree of diversity in the temporal dynamics of glutamatergic input onto MT cell dendrites in a glomerulus, over timescales involving a single inhalation and across multiple inhalations of odorant. We also directly compared presynaptic and postsynaptic excitation of MT cells using simultaneous, dual-color imaging of glutamate and calcium signals from MT cells in the same glomerulus. We found a very high correspondence between the dynamics and polarity of glutamatergic input and that of MT cell outputs as reflected by glomerular calcium signals. These results suggest that the complex spatiotemporal activity patterns thought to play a critical role in coding odor information may arise largely from diverse patterns of excitatory drive to MT cells.

## Materials and Methods

### Animals

Experiments were performed on male and female mice expressing Cre recombinase (Cre) in defined neural populations. Mouse strains used were: Pcdh21-Cre (Tg(Pcdh21-cre)BYoko), Gensat Stock #030952-UCD; OMP-Cre (Tg(Omp-tm4-Cre)Mom), JAX Stock #006668, Tbet-Cre (Tg(Tbx21-cre)1Dlc), JAX Stock #024507, and CCK-IRES-Cre (Tg(CCK-IRES-Cre)Zjh), JAX Stock #012706 (Haddad et al., 2013). Mice ranged from three to four months in age. Mice were housed up to four per cage and kept on a 12/12 h light/dark cycle with food and water available *ad libitum*. All procedures were conducted following the National Institutes of Health *Guide for the Care and Use of Laboratory Animals* and were approved by the University of Utah Institutional Animal Care and Use Committee.

### Viral vector expression

Viral vectors were obtained from the University of Pennsylvania Vector Core (AAV1 or 5 serotype, AAV.hSynap-FLEX.iGluSnFR and AAV.hSynap-FLEX.jRGECO1a), Addgene (AAV1 serotype, pAAV.hSynap-FLEX.SF-iGluSnFR.S72A, #106182), Howard Hughes Medical Institute Janelia Campus or Vigene (AAV1 or 5 serotype, pAAV.hSynap-FLEX.SF-iGluSnFR.A184V, pAAV.hSynap-FLEX.SF-iGluSnFR.A184S). Virus injection was done using pressure injections and beveled glass pipettes, as described previously (Rothermel et al., 2013; Wachowiak et al., 2013; Short and Wachowiak, 2019). For coinjection of jRGECO1a and SF-iGluSnFR.A184S, virus was either diluted 1:10 and injected in separate pipettes through the same craniotomy or mixed and coinjected via the same pipette. After injection, mice were given carprofen (rimadyl, 5 mg/kg, s.c.; Pfizer) as an analgesic and enrofloxacin (baytril, 3 mg/kg, i.m.; Bayer) as an antibiotic immediately before and 24 h after surgery. Mice were singly housed after surgery on ventilated racks and used 21–35 d after virus injection. In some mice, viral expression was characterized with *post hoc* histology using native fluorescence.

### *In vivo* two-photon imaging

Two-photon imaging in anesthetized mice was performed as described previously (Wachowiak et al., 2013; Economo et al., 2016). Mice were initially anesthetized with pentobarbital (50–90 mg/kg) then maintained under isoflurane (0.5–1% in O_2_) for data collection. Body temperature and heart rate were maintained at 37°C and ∼400 beats per minute. Mice were double tracheotomized and isoflurane was delivered passively via the tracheotomy tube without contaminating the nasal cavity (Eiting and Wachowiak, 2018). Two-photon imaging occurred after removal of the bone overlying the dorsal OB and stabilizing the brain surface with agarose and a glass coverslip.

Imaging in awake, head-fixed mice was performed through a chronic imaging window implanted over one dorsal OB. The imaging window consisted of a custom double coverslip with minimum diameter of 1.5 mm. Virus (500–750 nl) was injected at a depth of 250 μm at the time of window implant. Animals were acclimated to the imaging rig for at least one 30-min session the day before imaging. Imaging sessions lasted from 30–60 min/d, over the course of several days. The behavior and imaging apparatus has been described previously (Economo et al., 2016). Mice were naive to the odors at their first imaging session. Respiration (sniff) signals were obtained with an external flow sensor (FBAM200DU, First Sensor, AG) in front of the animal’s right nostril (Jordan et al., 2018), or by using a thermistor (MEAS-G22K7MCD419, Measurement Specialties) implanted in the right nasal bone (McAfee et al., 2016).

Imaging was conducted with a two-photon microscope (Sutter Instruments or Neurolabware) coupled to a pulsed Ti:Sapphire laser (Mai Tai HP, Spectra-Physics; or Chameleon Ultra, Coherent) at 920–940 nm and controlled by either Scanimage (Vidrio) or Scanbox (Neurolabware) software. Imaging was performed through a 16×, 0.8 N.A. objective (Nikon) and emitted light detected with GaAsP photomultiplier tubes (Hamamatsu). Fluorescence images were acquired using unidirectional resonance scanning at 15.2 or 15.5 Hz. For SF-iGluSnFR.S72A imaging, bidirectional scanning at 30 Hz was used to capture faster responses. For dual-color imaging, a second laser (Fidelity-2; Coherent) was used to optimally excite jRGECO1a (at 1070 nm) and emitted red fluorescence collected with a second PMT, as described previously (Short and Wachowiak, 2019).

### Odorant stimulation

In most experiments, odorants were presented as precise dilutions from saturated vapor (s.v.) in cleaned, desiccated air using a custom olfactometer under computer control, as described previously (Bozza et al., 2004; Economo et al., 2016). Odorants were presented for durations ranging from 2 to 8 s. Clean air was passed across the nostrils in between trials to avoid contribution from extraneous odorants in the environment. Odorants were prediluted in solvent (1:10 or 1:25 in mineral oil or medium chain triglyceride oil) to allow for lower final concentrations and then diluted to concentrations ranging from 0.3% to 1% s.v. Relative increases in concentration were confirmed with a photoionization detector (miniRAE Lite, PGM-7300, RAE Systems) 3 cm away from the flow dilution port. Estimated final concentrations of odorants used ranged from 0.01 to 300 ppm, depending on vapor pressure and s.v. dilution (Table 1). For experiments testing a larger panel of odorants we used a novel olfactometer design that allowed for rapid switching between odorants with minimal cross-contamination (Burton et al., 2019). Here, odorants were presented for 2 or 3 s, in random order from among a bank of 12 odorant cartridges, using 10-s interstimulus intervals. Odorant presentation used an eductor nozzle for optimal mixing in a carrier stream of filtered air. The end of the eductor was placed 5–7 cm away from the nose. With the configuration used, estimated dilutions of odorant were ∼1.5% s.v.; odorants were prediluted to achieve relatively sparse activation of dorsal glomeruli (Burton et al., 2019). Estimated final concentrations ranged from 0.01 to 16 ppm (Table 1).

**Table 1:** Odorants and concentrations used

Odor name	Odor number	Final estimated ppm (by experiment type)				
		ITA	1 Color	2 Color	Awake	Conc. 1x
Butyric acid	1		0.08	0.08		
2-Methylbutyric acid	2		0.03	0.03	0.03	
Valeric acid	3		0.03	0.03		
Hexanoic acid	5	0.2	0.01	0.01	0.01	0.05
Isovaleric acid	6		0.04	0.04		
2-Methylbuteraldehyde	9		2	2		
Trans-2-methyl-2-butenal	10		0.7	0.7	0.7	
Heptanal	11		0.7	0.7		
2-Methylvaleraldehyde	13		3	3	3	
Ethyl butyrate	19	10				2
Vinyl butyrate	20	9	2	2	2	3
Methyl valerate	21	8			5	2
Ethyl tiglate	22	2			0.6	0.6
Hexyl tiglate	23		1	1	1	
s-Methyl thiobutanoate	24		5	5		
Hexyl acetate	25		3	0.3		
Isopropyl tiglate	27		5	5	5	
Isoamylacetate	28		1	1		
2-Hexanone	29	10			12	3
Menthone	32	0.01				
2-Butanone	33		16	16		19
1-Hexanol	40		2	2		
Cyclohexylamine	43		15	15	15	
n-Methyl piperidine	45		1	1		
2-methoxy-3-methylpyrazine	51			1	1	
2-Isopropyl-3-methoxypyrazine	56		0.2	0.2		
Benzaldehyde	57	0.1				
Methyl benzoate	60	0.3				0.08
Isobutylthiazole	81			0.07	0.07	
3-Octen-2-one	102		4	4		
Trans-2-dodecanal	107		0.2			
Furfuyl mercaptan	110		0.7			
Isobutyraldehyde	136		5	5		30

Odorants and concentrations used in each experimental dataset. Odor number represents the internal reference number associated with each odorant, used in Figure 3. ITA; Figure 2, 1 color: iGluSnFR or SF-iGluSnFR imaged in anesthetized mice; Figure 3, 2 color: SF-iGluSnFR.A184S and jRGECO1a imaged simultaneously; Figures 6, 7. Awake: data from awake, head-fixed mice; Figures 5, 7, Conc (1×): lowest concentration of concentration series experiments; Figure 4, concentrations given as final estimated ppm, or the estimated vapor concentration of each odorant delivered to the animal, estimated from liquid dilution ratios, reported vapor pressures, and calibration of the odor delivery device.

### Data analysis

Image analysis was performed using custom software written in MATLAB (MathWorks). For display, odorant response maps were displayed using ΔF values rather than ΔF/F to minimize noise from nonfluorescent regions. Activity maps were scaled as indicated in the figure and were kept to their original resolution (512 × 512 pixels) and smoothed using a Gaussian kernel with σ of 1 pixel. For time-series analysis, regions of interest (ROIs) were chosen manually based on the mean fluorescence image and were further refined based on odorant response ΔF maps, then all pixels averaged within an ROI. All signals were upsampled to 150 Hz for analysis using the MATLAB pchip function. Time series were typically computed and displayed as ΔF/F, with F defined as the mean fluorescence in the 1–2 s before odorant onset.

**Figure 1.**
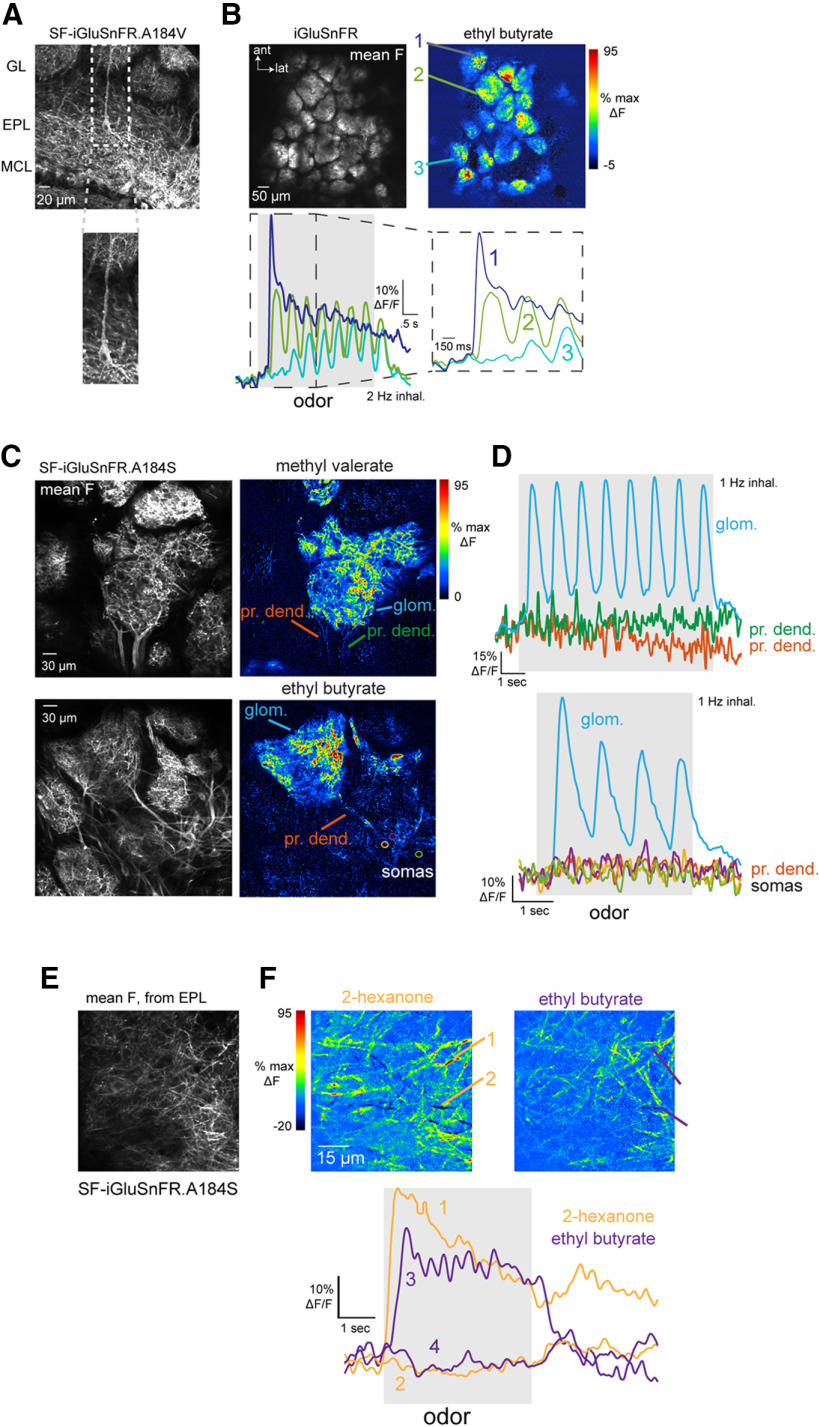
Characterization of iGluSnFRs as reporters of glutamatergic signaling onto MT cells. ***A***, Expression of SF-iGluSnFR in MT cells of the OB. Image shows confocal stack with expression in MT cells after injection of AAV2.1.hSynap.Flex.SF-iGluSnFR.A184V in the OB of a Tbet-Cre mouse. White arrows indicate a tufted cell with expression in the soma and primary dendrite extending to the glomerular layer (GL). MCL, mitral cell layer. ***B***, Top left, Mean fluorescence image taken *in vivo*, showing iGluSnFR expression in glomeruli after injection of AAV2.1.hSynap.Flex.SF-iGluSnFR into the OB a Pcdh21-Cre mouse. Top right, ΔF image showing responses to ethyl butyrate (mean of eight presentations, 2-Hz inhalation). Bottom, Traces showing odorant-evoked iGluSnFR signal in three glomeruli, with dashed region expanded at right. Note distinct temporal responses across successive inhalations for each glomerulus. ***C***, Examples of SF-iGluSnFR.A184S responses imaged at high zoom from different glomeruli. Images show mean F (left) and ΔF response maps (right) to ethyl butyrate, showing localization of iGluSnFR signal to the glomerular neuropil (glom.), with a lack of signal on primary dendrites (pr. dend.) or MT cell somata (somas). ***D***, Traces showing responses to ethyl butyrate taken from the glomerular neuropil, primary dendrite, and MT somata (locations indicated in ***D***), indicating negligible signal on the apical dendrite or somata. ***E***, SF-iGluSnFR.A184S expression imaged in vivo from Tbet-positive MT cell lateral dendrites in the superficial external plexiform layer (EPL). ***F***, Top: Odorant-evoked response maps imaged from the field of view in (a), showing responses to two odorants, 2-hexanone and ethyl butyrate. Bottom: Traces showing stronger excitatory and weak suppressive SF-iGluSnFR signals on different dendrites, evoked by each odorant.

For analysis of inhalation-linked dynamics, inhalation-triggered average (ITA) responses were generated by averaging each inhalation (delivered at 0.25 Hz) over a 70-s odorant presentation (17 inhalations averaged in total). Onset latencies, peak responses, and ITA durations (full-width at half-maximum) were defined as previously (Short and Wachowiak, 2019). To calculate decay time constants (Fig. 1), a single exponential was fitted from the peak of the unfiltered ITA trace and extending to the end of the trace. Time-to-peak values were calculated from unfiltered ITAs as the time from the inhalation start to the peak of the response.

For analysis of odorant-evoked dynamics over multiple sniffs, responses to three to eight presentations of odorant were averaged before analysis. Changes in response amplitude over time, or T_2_–T_1_/T_max_ were calculated as the difference in amplitude between the peak ΔF following the first (T_1_) and the second-to-last (T_2_) inhalation during odorant presentation, divided by the maximum ΔF during the 4-s odor presentation.

To analyze response patterns across the 23-odorant panel (Fig. 3), responses were averaged across three to four randomized presentations of each odorant. Responses were classified as having significant excitatory and/or suppressive components as follows. First, each averaged response for an ROI-odor pair was low-pass Gaussian filtered at 2 Hz (to test for excitatory responses) or 0.5 Hz (for suppressive responses) and z-scored using a baseline from 0.5 to 2 s before odorant onset, with z defined as the SD of the baseline period concatenated for all 23 odorant responses for each ROI. Peak excitation was measured as 95% of the maximum signal during the 3- or 4-s odorant presentation; suppressive responses were measured as the 15th percentile of all values in a time window from odorant onset to 500 ms after odorant offset. We then used a very conservative criterion for significance of z = ±7 SD for identifying significant excitatory or suppressive response components. This cutoff was chosen to yield a false positive rate of ∼1% (based on visual inspection of a subset of traces).

Glomerular tuning was characterized from response matrices thresholded according to these significance values, using a measure of lifetime sparseness (Schlief and Wilson, 2007):

$$S=\{1-[{\left(\sum^{}N_{j=1}r_{j}/N\right)}^{2}/\sum^{}N_{j=1}(r_{j}^{2}/N)\}/[1-(1/N)],$$

where N is number of odors and $r_{j}$ is the thresholded z-scored value for a given glomerulus of the response to odor j. Sparseness was calculated separately for excitatory and suppressive response components.

Time-dependent decorrelation of odorant response patterns across glomeruli (Figs. 3, 5) was calculated as previously described (Eiting and Wachowiak, 2020), using all glomeruli in a field of view without thresholding. Each odorant response was binned into five time bins (each of 387 ms) spanning the length of odorant presentation but omitting the first 65 ms to account for delays in odorant onset. Pearson’s correlation coefficient (*r*) was calculated using the response amplitudes across all ROIs in the first time bin as a vector, compared with the same vector taken from each subsequent bin. We calculated an overall correlation time series by computing the mean and SEM for all odorant responses, pooled across all fields of view.

For analysis of inhalation-linked timing of glutamate signals in awake mice, we restricted analysis to inhalations occurring at a minimum period of 200 ms (5-Hz respiration rate). Inhalation timing was detected from the external flow sensor using peak/trough detection and maximal slopes of the flow signal. Inhalation peak was defined as the trough of the external flow signal; inhalation onset was defined as the maximal slope of the transition from exhalation to inhalation. Because inhalation peak timing was a more robust measure, we generated ITA iGluSnFR signals based on inhalation peak. ITAs were generated by averaging the unfiltered optical signal taken from −0.3 s before to 0.4 s after inhalation. To avoid confounds of the airflow waveform or measurement method (airflow vs thermistor) in determining absolute ITA response latencies, we report the range of latencies relative to the median of a given set of measurements. Statistical tests were performed either in Origin (OriginLab Corp.), MATLAB (MathWorks) or R (version 3.3.2). Nonparametric tests were used in most cases; parametric tests were used only on datasets determined to be normally distributed. Summary statistics are reported as mean ± SD, unless otherwise stated. All measurement of response parameters was done using analysis code that was independent of treatment or comparison condition.

### Data and code availability

All data and analysis code are available from the corresponding author on request.

## Results

### Diverse inhalation-linked glutamate transients imaged from MT cell dendrites

The timing of odorant-evoked and inhalation-evoked MT cell spiking varies across MT cells and across odorants, and has long been hypothesized to play a role in coding odor information (Chaput, 1986; Schaefer and Margrie, 2007; Wilson et al., 2017). To assess the degree to which such variability is present at the level of glutamatergic input to MT cells, we targeted iGluSnFR and its second-generation variants to MT cells using Cre-dependent viral vectors and MT cell-specific Cre driver lines (Fig. 1*A*; Nagai et al., 2005; Haddad et al., 2013). We have previously shown, using the first-generation iGluSnFR, that each inhalation of odorant drives a transient of glutamate release onto juxtaglomerular interneuron processes in the glomerular neuropil (Brunert et al., 2016). We observed similar glutamate signals with iGluSnFRs expressed in MT cells, with different glomeruli showing glutamate transients with varying onset latencies and durations, and with distinct changes in the glutamate signal evoked by successive inhalations across a longer odorant presentation (Fig. 1*B*). In the superficial OB layers, glutamate signals appeared restricted to MT cell apical tufts within glomeruli, with negligible signals outside of the glomerulus or on MT cell primary dendrites or somata (Fig. 1*C*,*D*). Odorant-evoked glutamate signals were also detected along MT cell lateral dendrites in the external plexiform layer (EPL; Fig. 1*E*,*F*).

**Figure 2.**
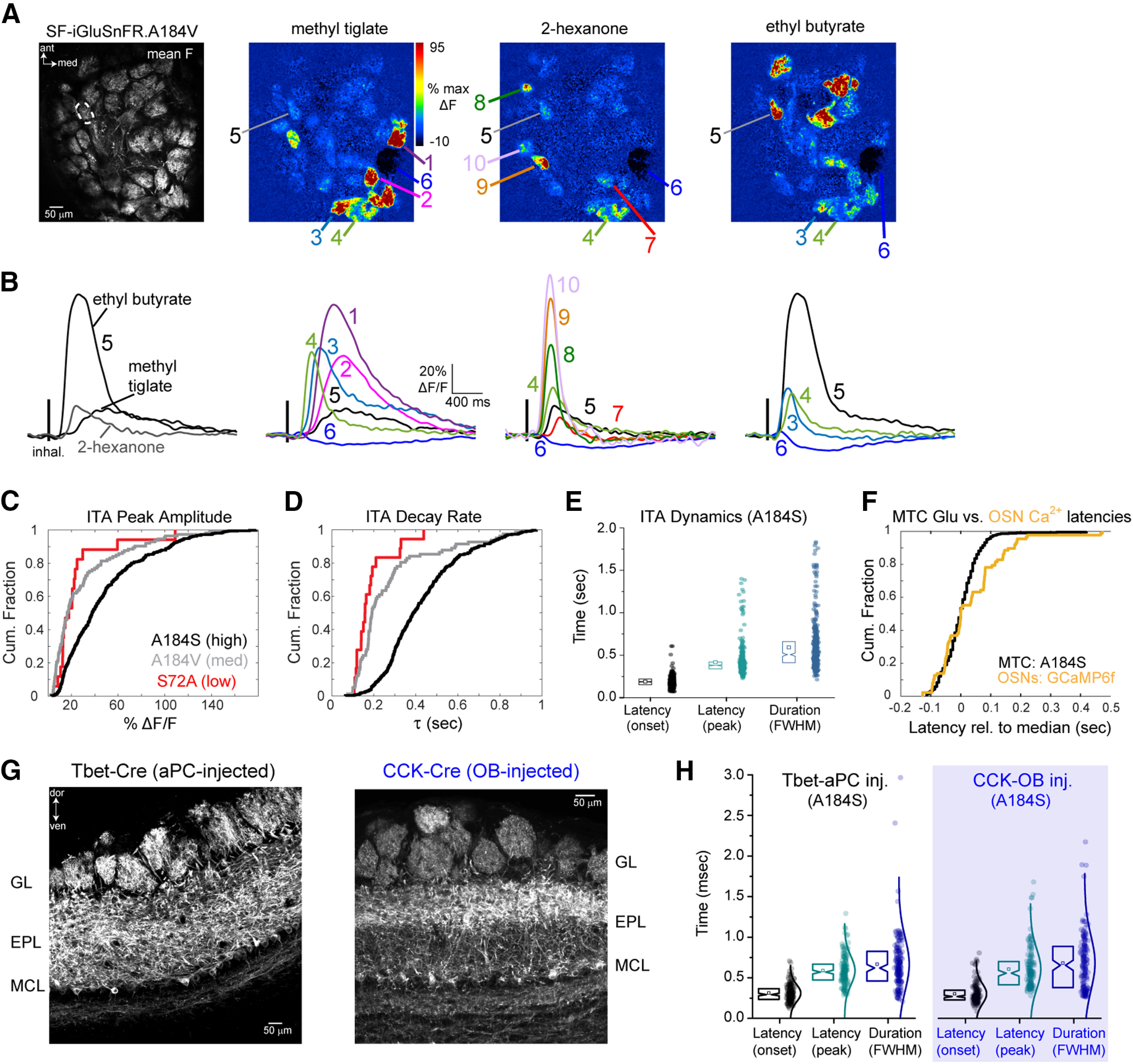
Glomerular glutamate signals across MT cell apical tufts show diverse inhalation-driven dynamics. ***A***, Left, Mean fluorescence image of SF-iGluSnFR.A184V expression imaged *in vivo*. Right, Inhalation-triggered ΔF response maps for three odorants. ***B***, ITA response traces for the glomeruli indicated in ***A***. Traces are averages across 17 inhalations. Left, ITAs from one glomerulus (ROI 5) responsive to three odorants, illustrating odorant-specific ITA dynamics. Right, ITAs from different glomeruli responsive to each of the three odorants, illustrating glomerulus-specific dynamics. ***C***, Cumulative fraction of %ΔF/F peak values across SF-iGluSnFR variants: black, A184S (high-affinity variant); gray, A184V (medium affinity variant); red, S72A (low-affinity variant). ***D***, Cumulative fraction of decay rates across SF-iGluSnFR variants: Same colors as in C. ***E***, Plot of ITA onset latency, peak latency, and duration (FWHM, full-width half-max) for all responsive glomerulus-odor pairs using SF-iGluSnFR.A184S. Notch box plots (left) of latency values (right). Notch: median, square: mean, box edges: 25th and 75th percentiles, *n* = 431 glomerulus-odor pairs, 5 mice. ***F***, Cumulative fraction of median-subtracted, ITA onset latencies for SF-iGluSnFR.A184S signals, compared with those measured from GCaMP6f expressed in OSN axon terminals (OMP-Cre x Rosa-GCaMP6f mice), showing similar range of latencies for both measurements**. *G***, Preferential iGluSnFR expression in sTCs versus mitral cells. Left, Confocal image of tissue section after Flex.AAV.SF-iGluSnFR injection into the superficial EPL of a CCK-IRES-Cre mouse. Note strongest expression in superficial EPL. Right, Similar image after Flex.AAV.SF-iGluSnFR injection into anterior piriform cortex in a Tbet-Cre mouse. ***H***, Identical distributions of onset latencies, times-to-peak, and durations (FWHM) of ITA waveforms imaged from CCK^+^ sTC and pcMT cell populations. Range of onset latencies (10th–90th percentiles): 195–451 ms (CCK^+^) versus 192–447 ms (pcMTs); times to ITA peak: 373–938 ms (CCK^+^) versus 367–828 ms (pcMTs); FWHM: 313–1058 ms (CCK^+^) versus 326–1007 ms (pcMTs); *n* = 174 and 160 glomerulus-odor pairs, respectively, from 3 mice each. All statistical comparisons, *p* > 0.05, Kolmogorov–Smirnov test.

We analyzed inhalation-linked dynamics of the glutamate signal across glomerulus-odor pairs by generating ITA responses from multiple inhalations at low frequencies (0.25–0.5 Hz; Díaz-Quesada et al., 2018; Short and Wachowiak, 2019). Most ITA transients consisting of a rapid rise and a slower decay to baseline. Odorant-evoked ITA dynamics varied substantially across glomeruli and odorants: glomeruli activated by the same odorant could respond with different onset latencies, rise times and durations, and the same glomerulus could show distinct ITA dynamics for different odorants (Fig. 2*A*,*B*). Inhalation occasionally elicited fluorescence decreases in a glomerulus, suggesting a phasic decrease in ongoing glutamate release (Fig. 2*B*, see ROI 6), likely because of the inhalation of clean air into the nasal cavity (Short and Wachowiak, 2019).

We measured ITAs across glomerulus-odor pairs using each of the three second-generation SF-iGluSnFR variants, A184S, A184V and S72A, which have higher, medium, and lower affinities for glutamate, respectively (Marvin et al., 2018). ITA response parameters were variable and highly overlapping across the three variants, but the high-affinity A184S variant showed a shift toward larger peak fluorescence changes and longer ITA decay times, consistent with earlier characterizations (Fig. 2*C*,*D*; Marvin et al., 2018; Armbruster et al., 2020). We made the most measurements with the medium-affinity and high-affinity variants because of their higher signal-to-noise ratios; across all glomeruli imaged with A184S, ITA onset latencies ranged from 113 to 253 ms (10th–90th percentiles), time to ITA peak ranged from 300 to 533 ms, and ITA durations ranged from 360 to 867 ms (Fig. 2*E*). Responses using the A184V variant were similar, with onset latencies ranging from 153 to 313 ms (10th–90th percentiles, 113 pairs from three mice), time to peak ranging from 313 to 727 ms, and ITA durations ranging from 260 to 1100 ms. This overlap suggests that variation in the dynamics of the SF-iGluSnFR signal imaged from MT cell tufts largely reflects the variable dynamics of glutamate signaling in the glomerulus, as opposed to kinetic differences of the reporter variant.

We compared the variability seen in the glutamate ITA with that from OSNs expressing the genetically-encoded reporter GCaMP6f, in response to the same odorants in separate mice. Despite the slower kinetics of GCaMP6f compared with iGluSnFR (Richter et al., 2018), the range of onset latencies (relative to median latency) was highly overlapping for GCaMP6f imaged from OSN terminals and iGluSnFR signals imaged from MT cell dendrites (Fig. 2*F*): median-subtracted onset latencies for OSNs (GCaMP6f) ranged from −91 to o189 ms (10th–90th percentiles; 87 glomerulus odor pairs, six mice), and −70 to 87 ms for MTs (iGluSnFR.A184S; 431 glomerulus-odor pairs, seven mice). These results suggest that variability in the onset times of glutamate transients onto MT cells after each inhalation can be largely accounted for by differences in the timing of spike bursts arriving at OSN presynaptic terminals.

We next asked whether the dynamics of glutamatergic input differ across MT cell subpopulations. Mitral cells and sTCs have distinct odorant response properties (Fukunaga et al., 2012; Igarashi et al., 2012). Many sTCs express the peptide neurotransmitter cholecystokinin (CCK; Seroogy et al., 1985), these neurons are strongly driven by monosynaptic input from OSNs (Sun et al., 2020), and we have previously shown that they have faster-onset and less diverse odorant-evoked responses than the general MT cell population (Economo et al., 2016; Short and Wachowiak, 2019; Eiting and Wachowiak, 2020). We compared glutamate dynamics onto CCK^+^ sTCs and piriform cortex-projecting MT cells (pcMTs), using Flex.AAV9.iGluSnFR.A184S virus injection into the superficial EPL of CCK-IRES-Cre mice or retrograde viral expression via injection into anterior piriform cortex of Tbet-Cre mice (Fig. 2*G*,*H*). The latter approach biases expression in mitral and deep tufted cells, with relatively little expression in sTCs (Rothermel et al., 2013). We found no difference in the dynamics of inhalation-linked glutamate transients onto CCK^+^ sTCs compared with pcMT cells, with both cell types showing a similar distribution of onset latencies, peak times and durations of the iGluSnFR ITA (Fig. 2*H*, see legend for summary statistics). These results indicate that reported differences in odorant-evoked response patterns in sTCs and pcMT cells do not arise from differences in the dynamics of their excitatory input.

### Diversity of glutamatergic signaling across multiple inhalations

**Figure 3.**
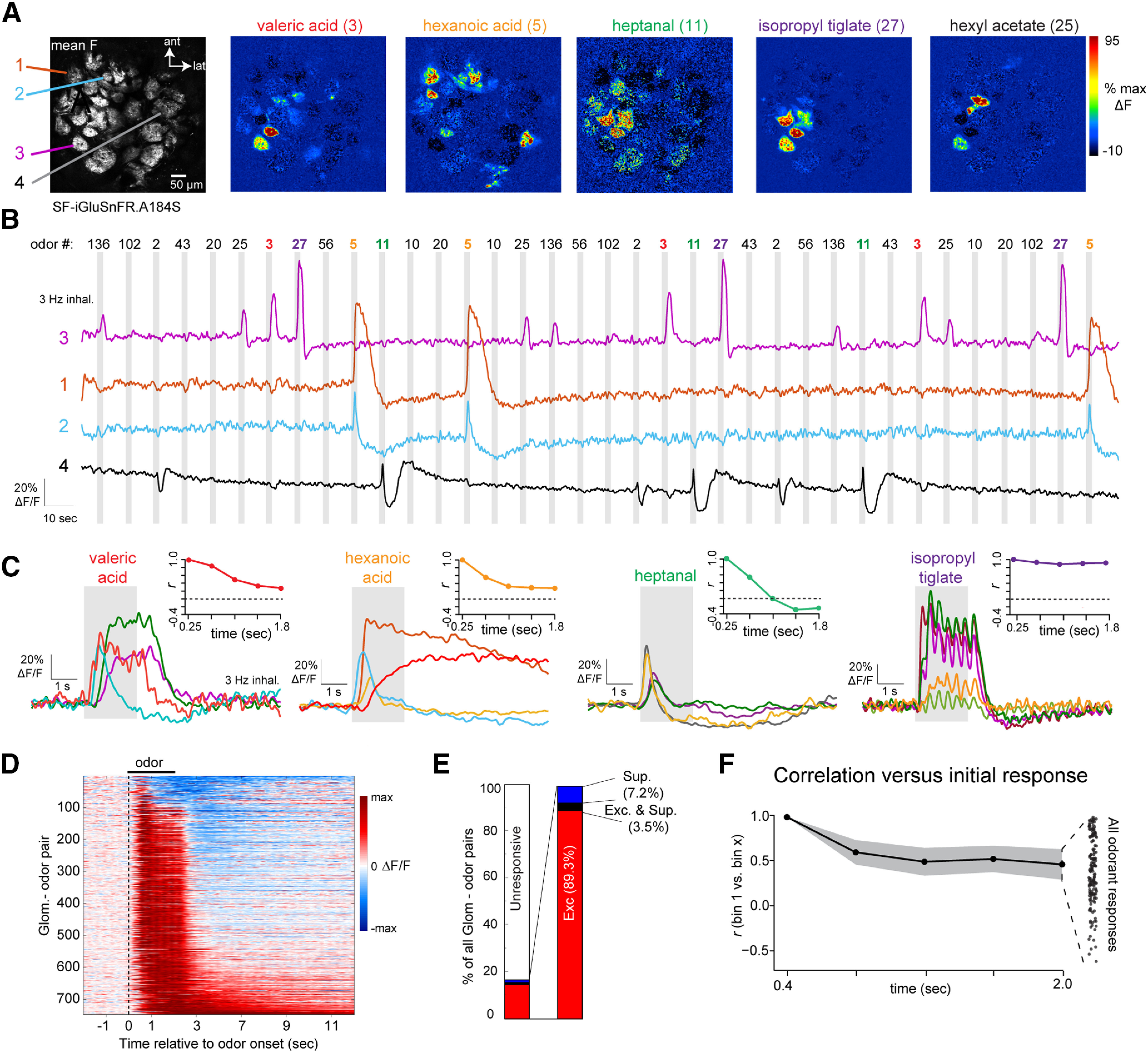
Glomerular glutamate signals show diverse temporal patterns across repeated inhalations of odorant. ***A***, Mean fluorescence (left) and ΔF response maps for five odorants imaged with SF-iGluSnFR.A184S (right). Odor numbers on the left side of the odorant map indicate odorant identification code for listed odor name (for reference to ***B***). Colored text indicates odorants whose responses are shown in panels ***B***, ***C***, ***F***. ***B***, Time series of SF-iGluSnFR.A184S signal from four glomeruli, showing continuous signal during three repetitions of 12 odorants, presented in random order. Shaded rectangle indicates time of odorant presentations (2-s duration). Signal is low-pass filtered at 0.5 Hz for display. ***C***, Average time course of odorant-evoked responses in different glomeruli responsive to four of the 12 odorants from ***B***. Insets show change in response pattern across the field of view from ***A*** over the duration of odorant presentation, expressed as Pearson’s *r* relative to the initial response in successive time bins of 387 ms (see text). Dotted line indicates *r* = 0. ***D***, Waterfall plot showing time course of odorant-evoked SF-iGluSnFR.A184S signal in all significantly-responding glomerulus-odor pairs (one pair per row), normalized to the peak ΔF/F for each pair. Suppressive responses are low-pass filtered at 0.5 Hz, and excitatory responses are low pass filtered at 2 Hz. ***E***, Proportion of excitatory (Exc.), suppressive (Sup) and biphasic (Exc. and Sup.) responses across the responsive population (right), as well as the entire population of glomeruli (left). ***F***, Change in glutamate response patterns during odorant presentation, summarized over all presentations. Black plot shows mean correlation over time, averaged across all odorant presentations. Shaded area indicates SEM. Dot plot at right shows correlation coefficients at the final time bin for all presentations.

To more fully investigate the temporal diversity in patterns of glutamate signaling onto MT cells, we imaged glomerular responses to 23 odorants in single sessions (see Table 1). Odorants were delivered at concentrations such that each odorant evoked relatively sparse responses across the imaging field (Fig. 3*A*); 16.5% of all glomerulus-odor pairs (748/4520; seven fields of view, three mice) showed responses that were significant according to a conservative criterion of ±7 SD deviation from baseline. Responsive glomeruli were narrowly tuned across the 23-odorant panel, with high values of lifetime sparseness for excited glomeruli (S = 0.91 ± 0.05, mean ± SD).

Temporal patterns of odorant-evoked glutamate signals were diverse but robust, occurring consistently over repeated presentations (Fig. 3*B*). The most common response pattern consisted of inhalation-linked transients that persisted over the 2-s presentation and returned rapidly to baseline (Fig. 3*C*, e.g., isopropyl tiglate). Other response patterns included transient increases that occurred immediately after odorant onset, prolonged increases that returned slowly to baseline after odorant offset, and slow, “facilitating” rises in glutamate (Fig. 3*C*, e.g., hexanoic acid). Response patterns varied for different glomeruli activated by the same odorant and for responses of the same glomerulus to different odorants (Fig. 3*C*), indicating that response patterns were neither a function of SF-iGluSnFR kinetics or expression levels in a particular glomerulus, nor a function of any particular odorant. Odorants also could evoke responses with a pronounced suppressive component (Fig. 3*B*,*C*), although these were relatively rare, with pure suppression seen in 7.2% (54/748) of glomerulus-odor pairs and biphasic (excitatory and suppressed) responses seen in 3.5% (26/748) of pairs (Fig. 3*D*,*E*).

A consequence of this diversity was that relative glutamate levels in different glomeruli varied over the odor presentation. To quantify this, we generated vectors consisting of time-binned glutamate signals (bin width, 387 ms) across all glomeruli in a field of view and correlated the vector in the first time bin with that from each successive time bin over the 2-s odor presentation. Across all odorant responses (*n* = 111 unique presentations), there was a substantial decorrelation beginning in the second bin (time bin 2 vs time bin 1: Δ*r* = 0.52 ± 0.32) and continuing to the last bin (time bin 5 vs time bin 1: Δ*r* = 0.39 ± 0.37), although the degree of decorrelation varied greatly for different odorants (Fig. 3*F*; see also Fig. 3*C*, insets). This decorrelation is qualitatively similar to that observed in patterns of MT cell spiking over repeated inhalations (Patterson et al., 2013; Díaz-Quesada et al., 2018; Eiting and Wachowiak, 2020), suggesting that time-dependent decorrelation can arise at the level of glutamatergic input to MT cells.

### Odorant concentration alters the dynamics of glomerular glutamate signals

**Figure 4.**
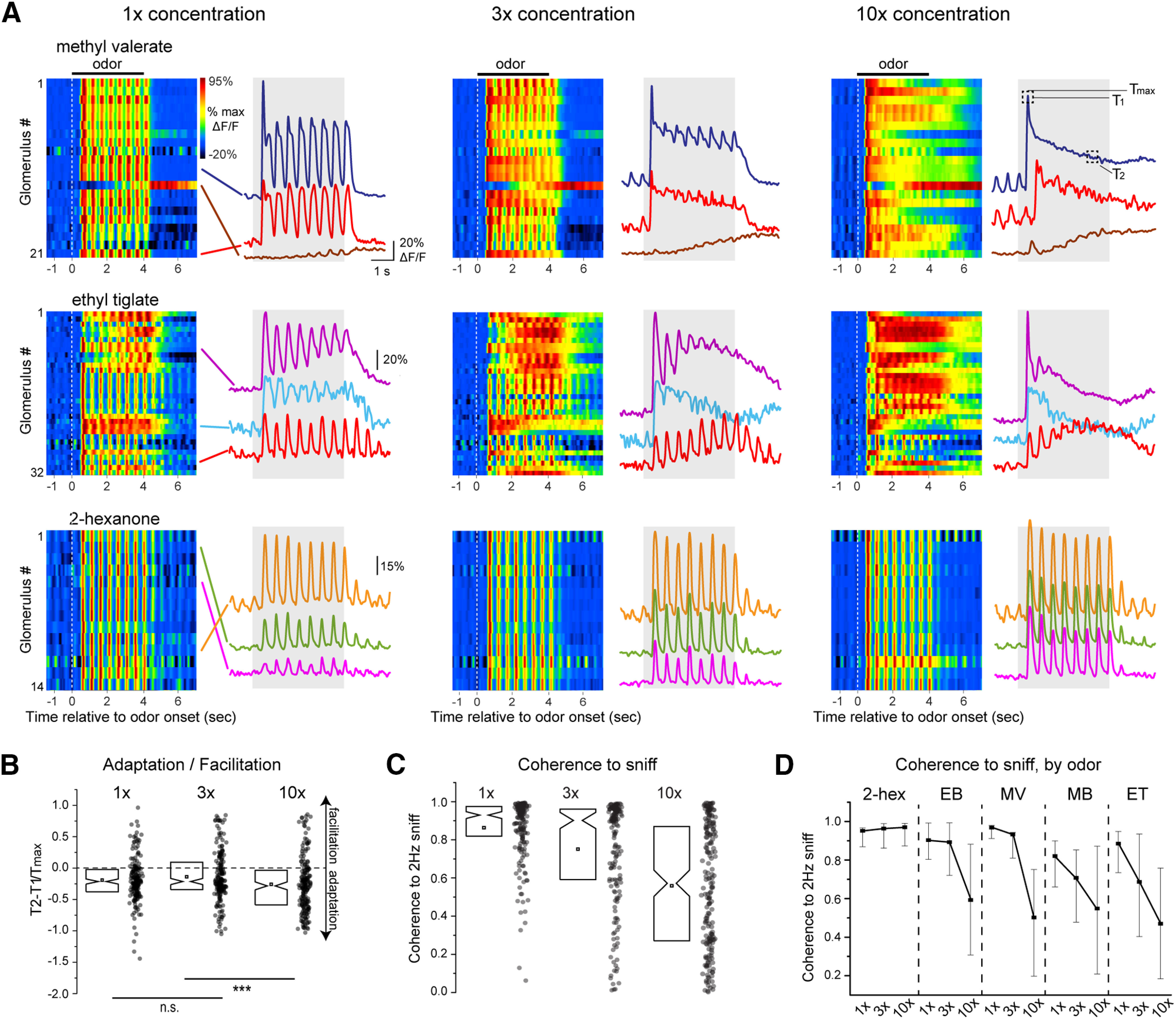
Odorant concentration systematically impacts glutamate signaling onto MT cells. ***A***, Waterfall plots (left) and traces (right) showing time course from select glomeruli showing original iGluSnFR signals in response to three concentrations of odorant for three different odorants (1×, 3×, 10× indicate relative concentration values; see text). All data are from the same experiment and field of view. Only glomeruli showing significant responses at all three concentrations are shown. Inhalation frequency, 2 Hz; odorant duration, 4 s. Signals are unfiltered. Glomerular identity is color coded with lines pointing to the traces. White dotted line indicates odor onset. T_1_, T_2_, T_max_ (10×, methyl valerate panel) indicates time points used for T_2_–T_1_/T_max_ measurements in ***B***. ***B***, Change in iGluSnFR response amplitude from beginning to end of odorant presentation (T_2_–T_1_/T_max_; see text), summarized for all analyzed glomerulus-odorant pairs (*n* = 189 glomerulus-odorant pairs from 4 mice). Notch: median, square: mean, box edges: 25th and 75th percentiles. Note high variability and slight but significant trend toward adaptation at all concentrations [one sample *t* test comparing T_2_–T_1_/T_max_; to zero, 1×: −0.19 ± 0.35 (mean ± SD), *p* = 2 × 10^−12^, 3×: −0.14 ± 0.40, *p* = 5 × 10^−6^, 10×: −0.26 ± 0.44, *p* = 8 × 10^−14^, *n* = 189 glomerulus-odorant pairs]. Lower bars indicate paired *t* tests between concentrations; ***p < 0.001; n.s., not significant. ***C***, Coherence of iGluSnFR signal to the 2-Hz inhalation frequency (see text), measured for the same glomerulus-odorant pairs in ***B***, showing high variability across glomeruli and a decrease in coherence at higher concentrations. ***D***, Coherence values as a function of concentration, showing a significant effect of odorant on concentration-coherence functions. Dot: median, error bars: quartiles.

Odorant concentration can impact the dynamics of glutamatergic signaling in the glomerulus, for example, by inducing rapid adaptation or sustained responses (Lecoq et al., 2009; Matsumoto et al., 2009). To address this, we presented a smaller number of odorants (three to five per experiment, *n* = 4 mice) at three concentrations spanning a 10-fold range, using 2-Hz inhalations and a 4-s odorant presentation. These experiments used first-generation iGluSnFR (Marvin et al., 2013) expressed in MT cells of Pcdh21-Cre mice. Changing concentration could dramatically alter the dynamics of the glutamate response (Fig. 4*A*). In many glomerulus-odor pairs, odorants evoked consistent inhalation-driven transients throughout odorant presentation at the lowest (1×) concentration, which changed to highly adapting responses at higher concentrations (Fig. 4*A*). In other glomeruli (even for the same odorants), increasing concentration appeared to recruit glutamate signals with repeated inhalations over the course of the presentation; this often included an increase in the tonic component of the glutamate signal (Fig. 4*A*, e.g., methyl valerate). Other glomerulus-odor pairs showed little adaptation or effects of concentration (Fig. 4*A*, e.g., 2-hexanone). We quantified these changes across concentration by calculating the normalized change in response amplitude from the beginning to the end of odorant presentation, as T_2_–T_1_/T_max_ (Fig. 4*A*, right, *B*). With this measure, negative values indicate adaptation while positive values indicate facilitation. There was high variability in this measure although adaptation was more common across the population, with significantly more negative values across all glomerulus-odor pairs (Fig. 4*B*; see legend for summary statistics). There was slightly more adaptation at medium (3×) concentrations compared with low (189 glomeruli, four mice, paired *t* test, *t* = −1.93, *p* = 0.055), and significantly more adaptation at the highest concentration (10×) compared with the medium (3×; paired *t* test, *t* = 6.62, *p* = 3.71 × 10^−10^).

A second effect of increasing odorant concentration was to reduce the degree to which glutamate signaling was modulated by each inhalation (Fig. 4*A*, see methyl valerate and ethyl tiglate responses). We quantified this effect by measuring the coherence of the iGluSnFR signal relative to a simulated 2-Hz inhalation pulse. Despite high variability in sniff coherence across glomerulus-odorant pairs, across all pair coherence decreased with increasing concentration (mean ± SD, 1×: 0.86 ± 0.16, 3×: 0.75 ± 0.28, 10×: 0.56 ± 0.32, one-way ANOVA, *F*_(3,552)_ = 2.54, *p* < 0.001; Fig. 4*C*). Notably, this effect varied depending on the odorant identity (two-way ANOVA, concentration x odorant: *F*_(8,552)_ = 3.87, *p* = 2.0 × 10^−4^). In particular, glomeruli responsive to 2-hexanone showing no loss of inhalation-linked coherence as concentration increased, while glomeruli responsive to the other tested odorants (all esters) showed a substantial loss of coherence (Fig. 4*D*). These odorant-specific differences may reflect differences in the kinetics of odorant sorption or clearance from the nasal epithelium. Overall, these results indicate that odorant concentration has systematic impacts on the dynamics of glutamatergic signaling onto MT cells, with a slight increase in adaptation and a substantial decrease in inhalation coupling as odorant concentration increases.

### Glomerular glutamate signaling shows diverse temporal dynamics in awake mice

**Figure 5.**
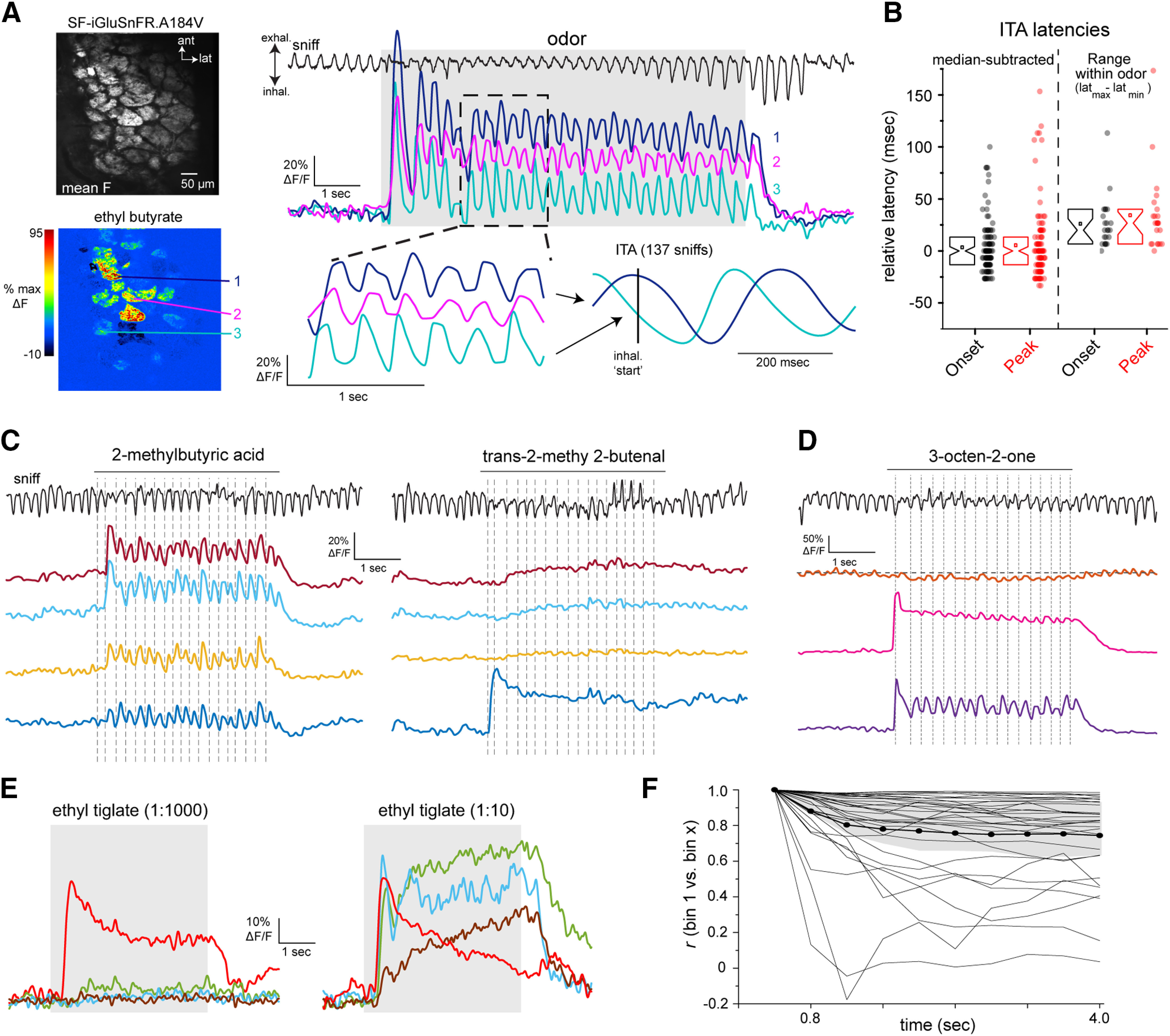
Diversity of glutamate dynamics imaged from MT cells in the awake mouse. ***A***, Top, Mean SF-iGluSnFR.A184V fluorescence. Bottom, ΔF response map (ethyl butyrate) from the dorsal OB of an awake, head-fixed mouse. Right, Glutamate signals from three glomeruli (shown in ***A***) during a single presentation of odorant (ethyl butyrate). Top trace shows respiration/sniffing as measured with an external flow sensor, with inhalation oriented downward. Lower left, snippet of signals from each glomerulus, illustrating temporal lag between different glomeruli and consistency of dynamics with each sniff. Signals are unfiltered. Lower right shows ITA waveforms for each glomerulus, generated across 137 sniffs over six presentations of ethyl butyrate. Vertical line indicates time of inhalation onset. ***B***, Left, Range of ITA onset (black) and peak (red) latencies, relative to median values, compiled across 108 glomerulus-odorant pairs (14 odorants, seven fields of view). Notch: median, box edges: 25th and 75th percentiles, square: mean values. Right, Spread of latency values seen across multiple responsive glomeruli imaged in the same field of view for the same odorant (defined as the difference between maximum (lat_max_) and minimum (lat_min_) latencies), for 17 unique odorant presentations. ***C***, SF-iGluSnFR.A184V signals imaged from four glomeruli from the same FOV showing responses to each of two odorants, showing distinct temporal response patterns across glomeruli and odorants. Traces are unfiltered and from a single presentation. Top trace shows respiration/sniffing for each trial; vertical dotted lines indicate inhalation peak for reference. ***D***, SF-iGluSnFR.A184V signals imaged from three glomeruli, taken from a separate field of view in the same mouse, showing distinct response patterns to a third odorant (3-octen-2-one). Note weak suppressive response in one glomerulus (top trace). ***E***, Increasing odorant concentration elicits changes in glutamate signal dynamics in awake mice. Traces show averaged responses (eight presentations) from four glomeruli in the same field of view to a low (1:1000 dilution) versus high (1:10) concentration of ethyl tiglate. Note that the response in the most sensitive glomerulus shows increased adaptation at the higher concentration, while other glomeruli show facilitating responses. ***F***, Changes in glutamate response patterns over the course of odorant presentation, calculated as described for Figure 3, in four awake mice, 33 odorant-FOVs (two mice, SF-iGluSnFR.A184V; two mice, SF-iGluSnFR.A184S). Plots of individual odorant responses are shown in gray, thick black line shows mean, shaded region is quartiles (25th and 75th percentiles). Note high variability in degree of decorrelation over time.

We next imaged iGluSnFR signals in awake, head-fixed mice while monitoring nasal airflow. Respiration frequencies were typically between 3 and 6 Hz (mean frequency per session, 4.4 ± 0.34 Hz, measured from five sessions), with occasional pauses and bouts of higher-frequency sniffing. Glutamate transients in awake mice were temporally diverse, similar to anesthetized mice. For many glomeruli, each inhalation at “resting” frequencies (defined as below 5 Hz) elicited a distinct glutamate transient (Fig. 5*A*,*B*). We measured inhalation-linked dynamics for glomerulus-odorant pairs showing significant respiratory modulation by constructing ITA waveforms as before, but using an external sensor to determine inhalation timing. ITA waveforms generated from these data were approximately sinusoidal (Fig. 5*A*, lower right), allowing for straightforward estimates of onset latency and time-to-peak of the glutamate signal relative to inhalation. Latency differences between glomeruli were apparent in single trials and were consistent across repeated inhalations of odorant (Fig. 5*A*). Overall, ITA onset latencies, compiled from four mice, varied over a range of 127 ms relative to the median latency across all glomerulus-odor ITAs in a session (SD of relative latencies = 25 ms, 14 odorants, 7 fields of view, 108 glomerulus-odorant pairs), ITA peak latencies spanned a range of 187 ms (SD = 34 ms; Fig. 5*B*). The range of latencies across different glomeruli activated by the same odorant (in the same field of view) was smaller, with a median range of only 27 ms for both onset and peak latencies (17 unique odorant presentations). As with artificial inhalation, these dynamics could vary across glomeruli and odorants.

For many glomerulus-odorant pairs, glutamate signals were not modulated by respiration but instead consisted of a tonic glutamate increase. The same glomerulus could show strong coupling to respiration for one odorant and a lack of respiratory coupling for another odorant (Fig. 5*C*). Similarly, different glomeruli responsive to the same odorant could show either strong or no respiratory coupling (Fig. 5*D*, indicating that the lack of coupling was not because of differences in respiration from trial to trial, nor was it because of differences in odorant stimulus profile. Odorants could also elicit decreases in the glutamate signal (Fig. 5*D*, top trace). Respiration-coupled and tonic glutamate signals were apparent to a similar degree in mice expressing SF-iGluSnFR.A184V or SF-iGluSnFR.A184S (two mice each).

Glutamate signals in awake mice also showed diverse odorant responses over multiple inhalations, including slowly facilitating and adapting response patterns (Fig. 5*C*,*D*). We also observed concentration-dependent changes in glutamate dynamics, with increasing odorant concentrations leading to stronger adaptation of glutamate signal for strongly-responsive glomeruli and recruitment of slowly-increasing responses in weakly-responsive glomeruli (Fig. 5*E*). Consequently, the relative patterns of glutamate signals across glomeruli changed from their initial pattern over repeated sniffs, and glutamate response patterns decorrelated more substantially for some odorants than others (Fig. 5*F*). Overall, these data indicate that the diversity in dynamics of glutamatergic inputs to MT cells observed in awake mice is qualitatively similar to that imaged with artificial inhalation.

### Correspondence between dynamics of glutamatergic inputs and MT cell postsynaptic activity

**Figure 6.**
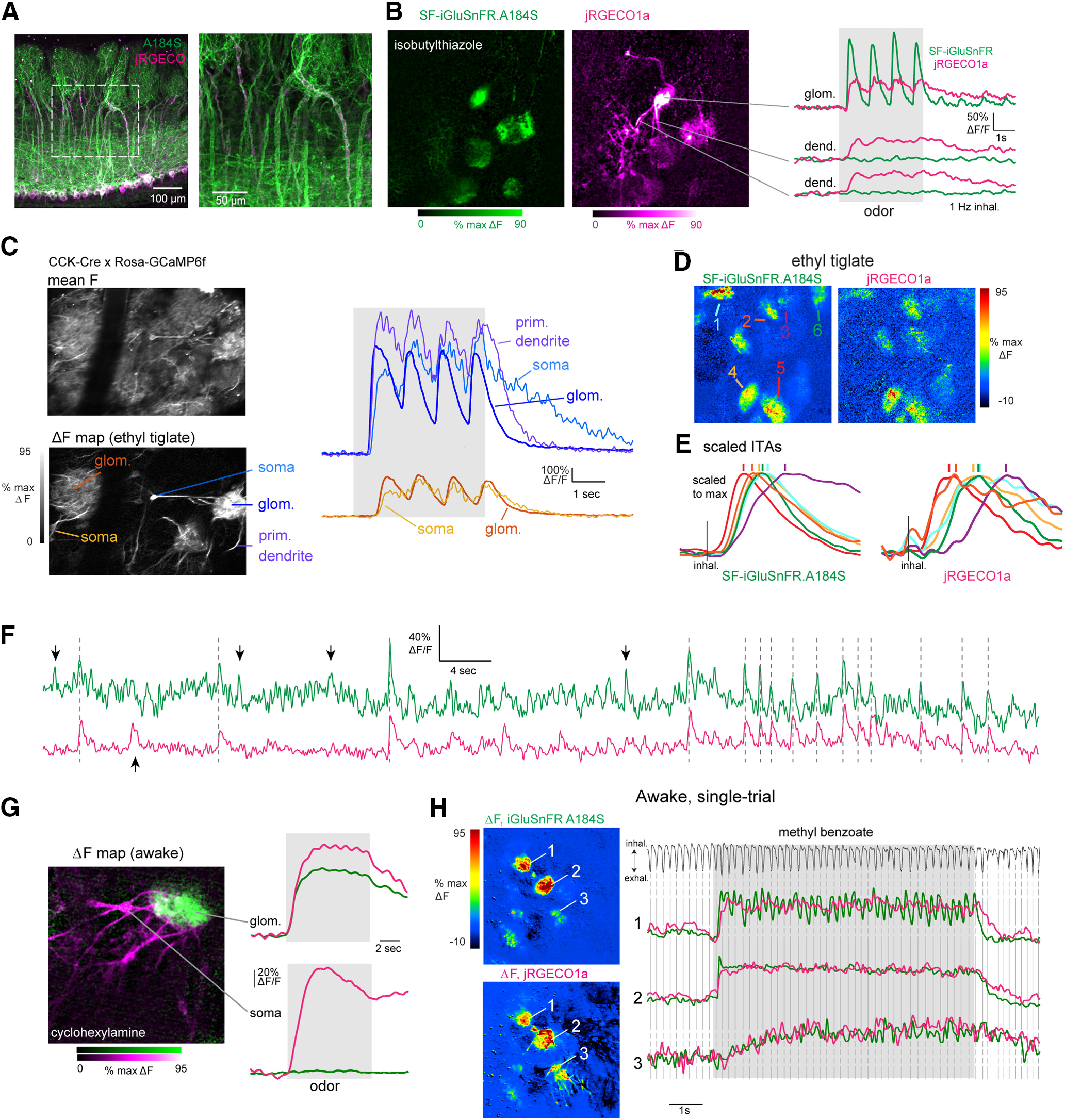
Dual-color imaging reveals high correspondence between presynaptic and postsynaptic signals in MT cells of the same glomerulus. ***A***, *Post hoc* confocal image showing coexpression of jRGECO1a (magenta) and SF-iGluSnFR.A184S (A184S, green) in MT cells of a Tbet-Cre mouse. ***B***, Dual-color two-photon imaging of SF.iGluSnFR.A184S and jRGECO1a signals from MT cells. Left, Odorant-evoked ΔF response maps for SF-iGluSnFR and jRGECO1a signals (magenta imaged simultaneously). Note jRGECO1a signal in dendrites of several MT cells exiting the central glomerulus, with SF-iGluSnFR signal confined to the glomerular neuropil. Right, Traces showing time course of the fluorescence signal in each channel (1-Hz inhalation). Top traces are from glomerular neuropil; lower traces are from dendrites outside of the glomerulus. ***C***, High correspondence in calcium signals imaged from different MT cell subcompartments. Left, Images show mean fluorescence and ΔF odorant response map (ethyl tiglate) for GCaMP6f signals imaged in a CCK-IRES-Cre: Rosa-GCaMP6f cross. Right, Overlaid traces showing time course of GCaMP6f signal from the neuropile of two glomeruli (blue, orange traces) and, for each, the soma of a tufted cell innervating each glomerulus. For the glomerulus on the right, the signal from the primary dendrite of a second tufted cell innervating the same glomerulus is also shown. Near-synchronous, inhalation-driven transients are seen in all compartments, with a slightly slower rise and slower decay in the somata. Latency differences between the two glomeruli are also present in their respective cells’ somata. ***D***, Pseudocolor odorant-evoked ITA response maps across the green (A184S) and red (jRGECO1a) channels. Arrows indicate ROIs with traces plotted in ***D***. ***E***, ITA traces taken from different glomeruli activated by the odorant in ***C***, with different onset latencies, times to peak and durations in different glomeruli. Left traces, SF-iGluSnFR.A184S. Right traces, jRGECO1a; traces from the same glomerulus are shown with the same color in each set. Each ITA trace is scaled to the same maximum. Vertical lines indicate peak time for the signal in each glomerulus. The relative order of peak times is the same for both signals. ***F***, Traces showing SF-iGluSnFR.A184V and jRGECO1a signals imaged simultaneously from a glomerulus, with high correspondence between spontaneously-occurring transients in the absence of odorant stimulation. Vertical lines mark transients seen in both signals; downward arrows mark transients seen in the green (A184S) but not red (jRGECO1a) channels; upward arrow marks transient seen in the red but not the green channel**. *G***, Dual-color imaging of SF.iGluSnFR.A184S and jRGECO1a signals from MT cells in the awake mouse. Left, Composite dual-color ΔF response map showing SF-iGluSnFR (green) and jRGECO1a (magenta) signals evoked by cyclohexylamine. Right, Traces showing fluorescence signal taken from the glomerular neuropile (top) and soma (bottom) of an innervating TC. Traces are average of eight presentations. ***H***, Trial-averaged ΔF response maps and traces for SF-iGluSnFR.A184S and jRGECO1a signals imaged from a single presentation of odorant in an awake mouse. Top trace shows respiration measured via external flow sensor. The SF-iGluSnFR signal clearly follows each inhalation in only one of the three glomeruli shown, while the jRGECO1a signal does not follow inhalations in any glomeruli.

While glutamatergic signaling in the glomerulus is the sole source of excitatory input to MT cells, inhibitory circuits and MT cell intrinsic properties can also shape patterns of MT cell excitation (Schoppa and Urban, 2003; Wachowiak and Shipley, 2006; Gire and Schoppa, 2009; Fukunaga et al., 2014; Burton, 2017; Ackels et al., 2020). To assess these relative contributions, we compared glutamate signals with calcium signals measured from MT cell apical tufts of the same glomerulus, imaged simultaneously. We co-expressed SF-iGluSnFR.A184S and the red calcium reporter jRGECO1a (Dana et al., 2016) in MT cells using viral co-injection into the OB of Tbet-Cre mice (Fig. 6*B*).

In response to odorant stimulation, jRGECO1a signals were apparent both in an activated glomerulus as well as in the primary dendrites of the MT cells innervating it, while SF-iGluSnFR signals were not apparent outside the glomerular neuropile (Fig. 6*B*,*E*). Because MT cell spikes back-propagate along the MT cell primary dendrite and invade the apical tuft, calcium signals imaged from the tuft can provide a reasonable proxy for patterns of MT cell spike output from each glomerulus (Bischofberger and Jonas, 1997; Chen et al., 1997; Charpak et al., 2001). Indeed, in separate calcium imaging experiments, we saw very high correspondence between odorant-evoked GCaMP6f signals imaged from the soma or primary dendrite of MT cells and those imaged from the neuropil of their parent glomerulus (Fig. 6*C*), consistent with our earlier reports (Economo et al., 2016).

In anesthetized mice, inhalation-linked transients were apparent in both signals at 1-Hz inhalation, although the jRGECO1a calcium signal decayed more slowly (Fig. 6*B*). Despite these kinetic differences, there was a close correspondence in the inhalation-linked dynamics of the two signals, with different glomeruli showing the same relative differences in the onset latency and time to peak of the SF-iGluSnFR and jRGECO1a ITA waveform (Fig. 6*D*,*E*). ITA onset latencies and peak times were highly correlated [Spearman’s rank correlation, ρ = 0.80 (latency); ρ = 0.78 (peak time)] and consistently delayed for the jRGECO signal [median (Q1 – Q3) Δlatency, 113 (80 −133) ms; median Δ peak time, 127 (80–180) ms; *n* = 17 glomerulus-odor pairs]. We also observed a close, although not perfect, correspondence in the occurrence of spontaneous transients in the SF-iGluSnFR and jRGECO1a signals (Fig. 6*F*). In awake mice, jRGECO1a signals showed little or no inhalation-linked modulation despite strong inhalation-linked transients in the SF-iGluSnFR signal (Fig. 6*G*,*H*), presumably because of the slower response kinetics of jRGECO1a.

**Figure 7.**
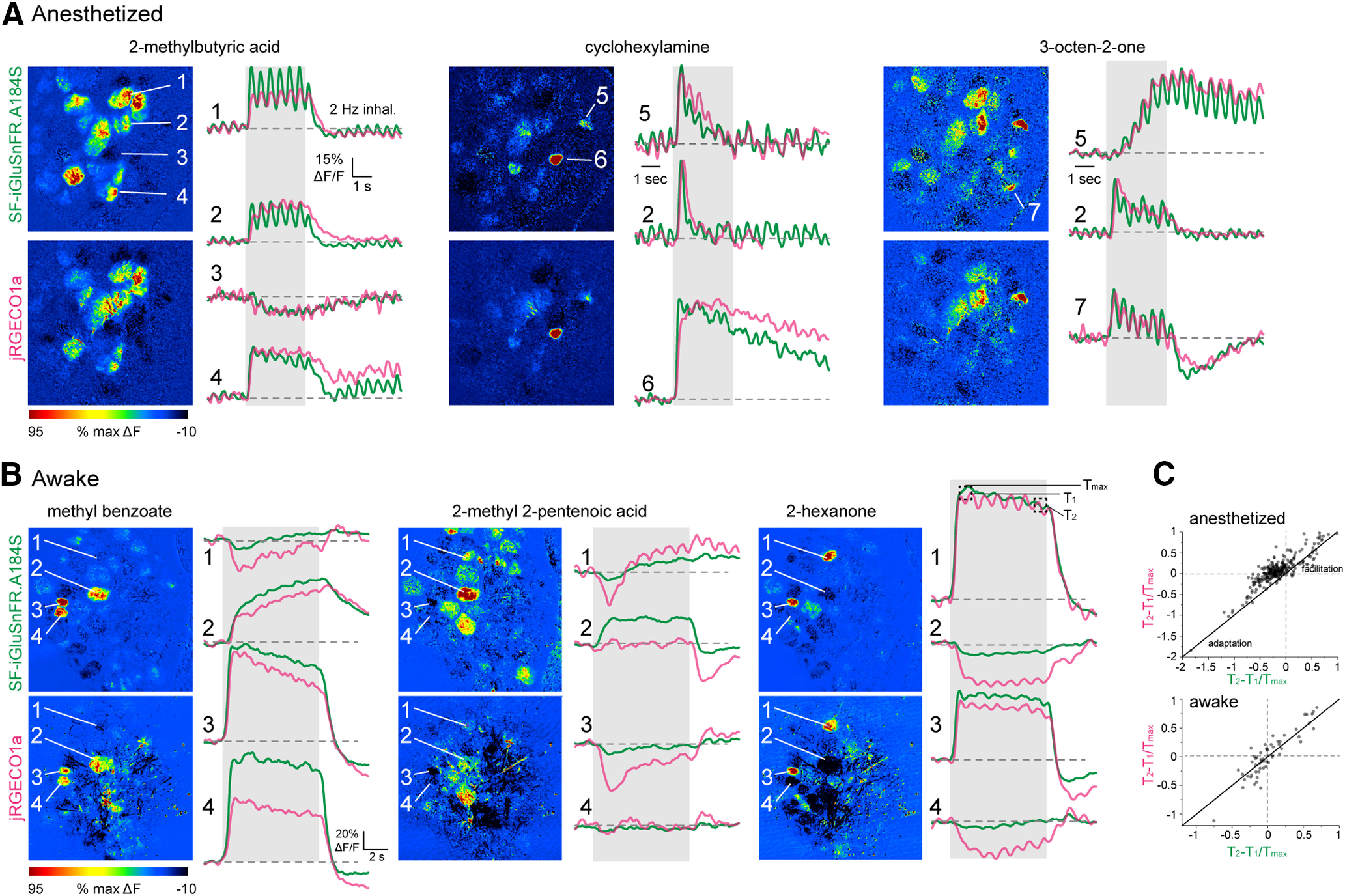
High correspondence between odorant-evoked temporal patterns of glutamate signaling and calcium activity in MT cells of the same glomerulus. ***A***, ΔF response maps (left) and trial-averaged SF-iGluSnFR.A184S and jRGECO1a signals imaged from select glomeruli for three odorants, illustrating high correspondence in slow response dynamics. Traces are average of four presentations in the same anesthetized mouse. Numbers indicate different glomeruli. Traces for cyclohexylamine and 3-octen-2-one are scaled to the same maximum. ***B***, ΔF response maps and trial-averaged SF-iGluSnFR.A184S and jRGECO1a responses taken from four glomeruli in response to three odorants in an awake mouse. Traces are average of 16 presentations. Note distinct response patterns for the same glomerulus in response to different odorants, but, with a few exceptions, similar response patterns for SF-iGluSnFR and jRGECO1a signals. ***C***, High correlation between T_2_ - T_1_/T_max_ values measured for excitatory SF-iGluSnFR and jRGECO1a responses in the same glomerulus in anesthetized (top) or awake mice (bottom).

To compare odorant-evoked response patterns over a timescale involving multiple inhalations, we first performed dual-color imaging in anesthetized mice using the same 23-odorant panel as for the single-color imaging (five fields of view from three mice). Overall, there was a striking degree of correspondence in response patterns of the two signals, with only rare exceptions (Fig. 7*A*). There was high concordance in glomeruli showing purely excitatory responses as measured with SF-iGluSnFR and jRGECO1a (using the conservative criterion of ±7 SD above baseline): 70% (273/387) of glomerulus-odor pairs with an excitatory SF-iGluSnFR signal also showed a significant jRGECO1a response, and 79% (273/346) of glomeruli with significant jRGECO1a increases showed excitatory SF-iGluSnFR signals. In awake mice, using a smaller odorant panel, only 46% (57/123) of glomerulus-odor pairs with a significant excitatory SF-iGluSnFR signal also showed jRGECO1a responses; however, 93% (57/61) of glomerulus-odor pairs with excitatory jRGECO1a responses also showed SF-iGluSnFR responses. Temporal response patterns were also highly correlated in awake mice (Fig. 7*B*). We quantified similarity in response patterns using the T_2_–T_1_/T_max_ index, which distinguished sustained, rapidly adapting, and facilitating responses. T_2_–T_1_/T_max_ values were highly correlated for glutamate and Ca^2+^ signals measured for the same glomerulus-odor pair, both in anesthetized and awake mice (Fig. 7*C*). Correlation coefficients for T_2_–T_1_/T_max_ values between SF-iGluSnFR and jRGECO1a responses were *r* = 0.87 in anesthetized mice (273 pairs, three mice) and *r* = 0.90 in awake mice (57 pairs, two mice). These results suggest that the evolution of MT cell activity patterns across inhalations largely follows that of glutamatergic input to these cells.

To infer how inhibitory circuits might shape MT cell spike output from glomeruli, we compared the prevalence of suppressive response components in the glutamate and Ca^2+^ signals. In anesthetized mice, suppressive responses were rare, and their prevalence may be underestimated from the relatively few repeat trials given (three to four per odorant) and the strict significance criteria we used. Nonetheless, jRGECO1a responses with suppressive components were detected significantly more frequently than SF-iGluSnFR responses, being present in 1.8% (72/3840) versus 0.7% (27/3840) of all glomerulus-odor pairs, and 17% versus 7% of all significant responses (χ^2^ statistic = 20.45, *p* = 6.0 × 10^−6^). In awake mice, suppressive responses were more prevalent, with a similar prevalence of suppressive components in both signals, being present in 21% (jRGECO1a: 92/446) versus 17% (SF-iGluSnFR: 78/446) of all glomerulus-odor pairs (χ^2^ statistic = 1.15, *p* = 0.28). Notably, suppressive jRGECO1a responses were very rarely seen in glomeruli showing excitatory SF-iGluSnFR signals, detected in only two of 72 suppressive jRGECO1a responses in anesthetized mice (2.7%) and five of 92 responses (5.4%) in awake mice; this prevalence is approaching the false positive rate of our measure of response significance. This result is surprising given predictions from slice experiments that weak excitatory input can lead to MT cell suppression by driving feedforward inhibition (Gire and Schoppa, 2009; Cleland and Linster, 2012; Gire et al., 2019). However, it is consistent with a model in which interglomerular inhibition suppresses MT cell spiking in a small fraction of glomeruli (Whitesell et al., 2013; Economo et al., 2016; Liu et al., 2016). Overall, the high correspondence between presynaptic glutamate and postsynaptic calcium signals suggests a large role for feedforward excitation in shaping MT cell response diversity.

## Discussion

Temporally complex patterns of excitation and inhibition among principal cells of the OB (MT cells) play important roles in coding olfactory information (Chaput, 1986; Schaefer and Margrie, 2007; Patterson et al., 2013; Díaz-Quesada et al., 2018). Such patterns have largely been thought to arise from OB circuits or intrinsic properties of MT cells themselves (Balu and Strowbridge, 2007; Gire and Schoppa, 2009; Shao et al., 2012; Fukunaga et al., 2014; Geramita and Urban, 2017). By directly imaging glutamate signaling onto MT cell dendrites of OB glomeruli, we observed temporally complex patterns of excitatory signaling both on the timescale of a single respiratory cycle and over a slower timescale involving repeated samples of odorant. Furthermore, simultaneous imaging of presynaptic glutamate and postsynaptic calcium from MT cells in the same glomerulus showed high correspondence in the dynamics of glutamatergic input and MT cell output. These results suggest a model of OB circuit function in which the dynamics of excitatory input to glomeruli may drive much of the observed diversity of MT cell responses that underlies odor representations *in vivo*.

This interpretation is subject to several caveats, including the suitability of iGluSnFR for reporting the dynamics of glutamate signaling and its potential to perturb these dynamics. Recent characterizations suggest that the iGluSnFRs are well suited to characterizing glutamatergic signaling in the OB *in vivo*, with intrinsic kinetics (rise and decay times of <10 to ∼20 ms, respectively; Marvin et al., 2013; Armbruster et al., 2016; Pinky et al., 2018) that are much faster than the dynamics of odorant-evoked excitatory input to MT cells (EPSP rise and decay times of ∼100 and 200 ms, respectively; Cang and Isaacson, 2003). iGluSnFR expression can prolong the decay of glutamate transients by competing with glutamate transporters (Armbruster et al., 2020), and decay rates are slower (∼140 ms) for the high-affinity SF-iGluSnFR.A184S (Marvin et al., 2018; Jensen et al., 2019). While we did find that decay times skewed longer for ITA responses measured with the high-affinity A184S variant, their distribution largely overlapped with that of the medium-affinity A184V. Furthermore, the degree of respiratory modulation as well as overall diversity in slow iGluSnFR signal dynamics appeared similar for the two variants. Thus, the SF-iGluSnFR signal imaged from MT cell apical tufts is a reliable reporter of the variable dynamics of glutamate signaling in the glomerulus *in vivo*.

Additional caveats in interpreting iGLuSnFR signal diversity arise from the multiple sources of glutamate signaling within the glomerulus, which includes OSN inputs, feedforward disynaptic excitation mediated by ET cells, and MT cells themselves, via dendritic release of glutamate from their apical tuft. Each of these pathways may have distinct dynamics of glutamate release during odorant stimulation, and each is subject to modulation by inhibitory OB circuits. The present data allow for some predictions to be made about the relative contributions of these pathways to excitatory dynamics.

Our data suggest that a major source of diversity in excitatory inputs to MT cells is OSNs themselves. OSN inputs to the OB respond with different latencies relative to inhalation in a glomerulus-specific and odorant-specific manner (Spors et al., 2006; Iwata et al., 2017; Short and Wachowiak, 2019; Ackels et al., 2020), and we found a close match in the range of response latencies for SF-iGluSnFR signals and those measured from OSN presynaptic terminals using Ca^2+^ reporters. We also saw variation in inhalation-linked glutamate dynamics over a slower timescale that could include adaptation, facilitation, or biphasic response patterns. Such complex slow dynamics can arise at the level of OSN activation because of phenomena such as enhanced responses as odorant concentration at the nasal epithelium is increased during repeated sampling, or reduced responses because of adaptation of OSNs or depression of OSN transmitter release (Murphy et al., 2004). These effects could underlie the nonlinearities in glutamate signal seen over the course of odorant presentation and as a function of odorant concentration. In addition, each of these effects may be amplified or gated by increasing sampling frequency, such as occurs during active sniffing.

Glutamate release from OSNs is subject to modulation by intraglomerular inhibitory circuits, via GABA_B_-mediated presynaptic inhibition (Aroniadou-Anderjaska et al., 2000; McGann et al., 2005; Wachowiak et al., 2005). However, we have shown previously that presynaptic inhibition contributes little to the temporal dynamics of calcium in OSN presynaptic terminals following a single inhalation or as a function of inhalation frequency (Pírez and Wachowiak, 2008), and that GABA_B_ receptor blockade does not alter the inhalation-linked dynamics of odorant-evoked iGluSnFR signals (Brunert et al., 2016). Thus, presynaptic, inhibitory glomerular circuits may shape the gain but likely not the temporal dynamics of glutamatergic transmission from OSNs to MT cells *in vivo*.

A second major source of diverse glutamate signaling onto MT cells is feedforward disynaptic excitation mediated by ET cells (Hayar et al., 2004b; De Saint Jan et al., 2009; Najac et al., 2011). ET cell-mediated glutamatergic drive could underlie the longer-duration glutamate transients seen in our data, as well as facilitation of glutamate signals over repeated samples, as ET cells become progressively entrained to the inhalation rhythm (Hayar et al., 2004a). Likewise, gating of ET cell excitation by inhibitory juxtaglomerular circuits may underlie short-duration odorant-evoked glutamate transients or the suppressive components observed in a minority of responses (Gire and Schoppa, 2009; Whitesell et al., 2013; Banerjee et al., 2015; Liu et al., 2016).

Dendritic release of glutamate from MT cells themselves may also contribute to the diverse iGluSnFR signals observed here, such glutamate “autoreception” is driven by invasion of somatic action potentials into the dendritic tuft and can exhibit facilitation or depression during sniff-like mitral cell activity in OB slices (Schoppa and Westbrook, 2002; Christie and Westbrook, 2006; Pimentel and Margrie, 2008). A strong contribution from such dendritic release could boost the apparent correspondence between iGluSnFR and jRGECO signals imaged from MT cell apical tufts. However, OB slice experiments suggest that the magnitude of dendritic glutamate release is likely small relative to that of feedforward glutamatergic drive arising from OSNs and ET cells (Schoppa and Westbrook, 2002; Pimentel and Margrie, 2008). Ultimately, pharmacological or genetic manipulation of these circuit elements – in combination with glutamate imaging, will be critical in determining the relative contribution of each pathway to excitatory MT cell drive *in vivo*.

Several aspects of these results have implications for how olfactory information is represented during natural odor sampling. First, in both awake and anesthetized mice, the relative magnitudes of glutamate signal across different glomeruli changed over the course of an odor presentation, suggesting that odorant representations evolve rapidly during repeated sampling of odorant. This result is consistent with recent findings of dynamic odor representations at the level of MT cell spiking (Patterson et al., 2013; Díaz-Quesada et al., 2018). Second, we found that a substantial fraction of odorant-evoked glutamate signals showed minimal or no respiratory patterning, instead showing tonic increases in glutamate when odorant was present. Assuming a similar fraction of MT cells fail to show respiratory patterning in their spike timing in awake mice, this result has implications for theories of odor identity or intensity coding that are based on the timing of MT cell spikes within the breathing cycle (Chaput, 1986; Schaefer and Margrie, 2007; Wilson et al., 2017; Li et al., 2020). Third, we found that the temporal evolution of glutamate responses as well as the degree of inhalation-linked patterning varies with odorant identity and concentration as well as glomerular identity. Comparable richness in response kinetics have been well characterized in *Drosophila* OSNs, where such features are attributed to odorant receptor identity (Mathew et al., 2013).

We observed a close (but not perfect) correspondence between the dynamics of glutamatergic input to MT cells and those of postsynaptic Ca^2+^ signals, imaged simultaneously. Interpreting these results is somewhat limited by the dynamic range and relatively slow kinetics of jRGECO (and GCaMPs) compared with that of MT cell spiking. However, even on a slower timescale, we saw no evidence for widespread inhibition surrounding glomeruli receiving excitatory inputs, as predicted by models of center-surround or global inhibition (Luo and Katz, 2001; Banerjee et al., 2015). We also observed little evidence for suppression of MT cell output in glomeruli receiving weak excitatory inputs, as predicted from models of feedforward inhibition (Gire and Schoppa, 2009; Cleland and Linster, 2012; Gire et al., 2019), although in awake mice we frequently observed iGluSnFR signals that were not accompanied by jRGECO responses in the same glomerulus. Thus, feedforward inhibition may prevent odorant-evoked increases in MT cell spiking without suppressing ongoing activity. Overtly suppressive MT cell responses were sparse and often co-occurred with a reduction in the glutamate signal to below baseline levels, consistent with MT cell suppression reflecting a reduction in excitatory drive. This reduction could be because of adaptation of OSN inputs (Lecoq et al., 2009), inhibition at the receptor level (Inagaki et al., 2020; Zak et al., 2020), or inhibition of ongoing glutamatergic drive from ET cells (Gire and Schoppa, 2009; Shao et al., 2012).

Finally, these results demonstrate the utility of iGluSnFR and its second-generation “SF”-variants to robustly report glutamate signaling onto MT cells with high signal-to-noise ratios and high temporal fidelity. The faster onset and decay times of SF-iGluSnFR relative to GCaMP or jRGECO allowed us to better monitor neural dynamics in the frequency range of respiration or sniffing in behaving mice, which ranges from ∼2 to 10 Hz (Wesson et al., 2008). We were able to observe clear glutamate transients from each inhalation in awake, head-fixed mice at sniff frequencies up to and, in some cases, exceeding 5 Hz. This improved temporal fidelity will be important in addressing hypotheses about determinants of the relative timing of excitatory inputs to MT cells in different glomeruli and the role of this timing in odor coding. Further use of iGluSnFRs and other transmitter-specific reporters could help disentangle the contributions of excitation and inhibition to shaping dynamic odor representations *in vivo*.

## Synthesis

Reviewing Editor: Arianna Maffei, SUNY Stony Brook Decisions are customarily a result of the Reviewing Editor and the peer reviewers coming together and discussing their recommendations until a consensus is reached. When revisions are invited, a fact-based synthesis statement explaining their decision and outlining what is needed to prepare a revision will be listed below. The following reviewer(s) agreed to reveal their identity: NONE. Note: If this manuscript was transferred from JNeurosci and a decision was made to accept the manuscript without peer review, a brief statement to this effect will instead be what is listed below.

The authors thoroughly addressed the reviewers' comments, clarifying their findings and adding details about experimental design and data analysis.
